# Intraoperative herniation of an L5-S1 disc during microdiscectomy and transforaminal lumbar interbody fusion: a case report

**DOI:** 10.1186/s13256-015-0766-6

**Published:** 2015-11-27

**Authors:** Connor D. Berlin, Thirumoorthi V. Seshan, John M. Abrahams, Ezriel E. Kornel

**Affiliations:** 1 Weill Cornell Brain and Spine Center, Department of Neurological Surgery, New York-Presbyterian Hospital/Weill Cornell Medical Center, 525 East 68 Street, Box 99, New York, NY 10065 USA; 2 Neuro Alert Monitoring Services, 244 Westchester Avenue Suite 316, West Harrison, NY 10604 USA; 3 Neurosurgery, New York Medical College, 40 Sunshine Cottage Road, Valhalla, NY 10595 USA; 4 Brain and Spine Surgeons of New York, 244 Westchester Avenue Suite 310, White Plains, NY 10604 USA

**Keywords:** Discectomy, Complication, Intraoperative, Reherniation, Intraoperative neurophysiological monitoring, Somatosensory evoked potentials

## Abstract

**Introduction:**

We report the progression of an intraoperative L5-S1 lumbar disc herniation that occurred during a routine microdiscectomy and transforaminal lumbar interbody fusion, which, to the best of our knowledge, has never been previously reported in the literature. The objective of this report is to bring to light the possibility of a lumbar disc herniating intraoperatively, and to demonstrate that accompanying neurologic involvement can be detected and subsequently addressed with the aid of neurophysiologic monitoring.

**Case presentation:**

A 36-year-old African American woman, who had previously undergone minimally invasive microdiscectomy for a right L5-S1 herniated nucleus pulposus with full recovery, presented with a large reherniation of the L5-S1 disc on the right side. During her operation, while a tap was followed into the L5 left pedicle, there was a sudden profound spasm of our patient’s legs and back that lasted for the duration of 15 seconds, culminating in the loss of all somatosensory evoked potentials in our patient’s lower extremities. Exploration of this previous microlaminotomy site revealed a massive disc extrusion protruding through the microlaminotomy. Immediate removal of this extruded disc material restored all somatosensory evoked potentials and our patient awoke with no neurologic deficits.

**Conclusions:**

An intraoperative disc herniation in the lumbar spine, though very rare, can occur and can result in neurologic compromise as evidenced by the loss of somatosensory evoked potentials. By identifying the event, it can be remedied by evaluating the disc visually, removing extruded fragments and decompressing nerve roots with recovery of somatosensory evoked potentials and normal neurologic function postoperatively. If neurophysiological monitoring shows there is a sudden loss of response, then consideration should be given to the possibility of an acute intraoperative herniation.

## Introduction

Surgical intervention may be indicated when nonsurgical management of a symptomatic herniated lumbar disc, including physical therapy and epidural steroid injections, fails. A small percentage of patients who undergo primary lumbar discectomy develop symptoms of failed back surgery syndrome postoperatively, with recurrent lumbar disc herniation as a leading cause [1]. Current literature indicates that the chances of recurrent lumbar disc herniation following a primary discectomy is 5–15 % [2, 3, 4, 5, 6]. Transforaminal lumbar interbody fusion (TLIF) is a well-accepted method for the treatment of recurrent lumbar herniation, demonstrating improved clinical outcome and a low surgical complication rate [7].

In the case described, a 36-year-old woman undergoing a microdiscectomy and TLIF for a recurrent L5-S1 disc herniation developed acute further herniation of this disc intraoperatively, as evidenced by the loss of somatosensory evoked potentials (SSEPs). To the best of our knowledge, such a neurologic complication during surgery has never been previously described in the literature.

## Case presentation

**Fig. 1:**
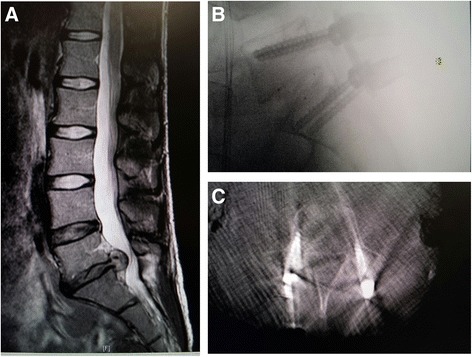
Clinical diagnosis and surgical treatment. Preoperative sagittal magnetic resonance imaging (MRI) depicting the L5-S1 disc herniation immediately before surgery. The herniated disc can clearly be seen protruding into the spinal cord (**a**). Postoperative sagittal computed tomography (CT) scan immediately following surgery and transverse X-ray image of screws and interbody device at L5-S1 1 week later, both demonstrating accurate screw placement into the vertebral pedicles. It is clear that there was no protrusion of the screws into the spinal cord, and the pedicle was therefore properly tapped (**b** and **c**)

A 36-year-old African American woman with a history of occasional backaches for years had developed right leg pain for 2 months that was profound and incapacitating. Our patient had previously undergone minimally invasive microdiscectomy for a right L5-S1 herniated nucleus pulposus and made a full recovery, with no significant back pain and slight right leg pain (1–2/10). She had no symptoms for 6 weeks until she presented again complaining of difficulty walking and severe lower back pain radiating to her right lower extremity. Her neurologic examination demonstrated positive straight leg rising at 30° on the right, decreased pinprick over the lateral aspect of the right foot, and absent ankle jerk on the right. Repeat magnetic resonance imaging (MRI) showed a large reherniation of the same L5-S1 disc on the right side (Fig. 1a).

Due to worsening symptomatology and the size of the herniation, our patient was inclined to proceed with surgery. An L5-S1 secondary minimal-access endoscopic microdiscectomy and TLIF with O-arm computed tomography (CT) imaging and frameless stereotaxic guidance was performed. The approach to the discectomy was to be done at L5 on the right, so a decision to first place pedicle screws on the left side percutaneously, followed by a right side discectomy and meticulous disc fragment extraction, then subsequent placement of screws on the right side, was made.

Intraoperative neurophysiological monitoring (IONM) was performed with SSEPs of bilateral tibial nerves, free-run electromyography (EMG), and triggered EMG via pedicle screw stimulation. The tibial nerves were stimulated with surface electrodes at the level of the medial malleolus of the ankles. EMGs were monitored with disposable needle electrodes in the following muscles of both lower extremities: vastus medialis, tibialis anterior, abductor hallucis.

**Fig. 2:**
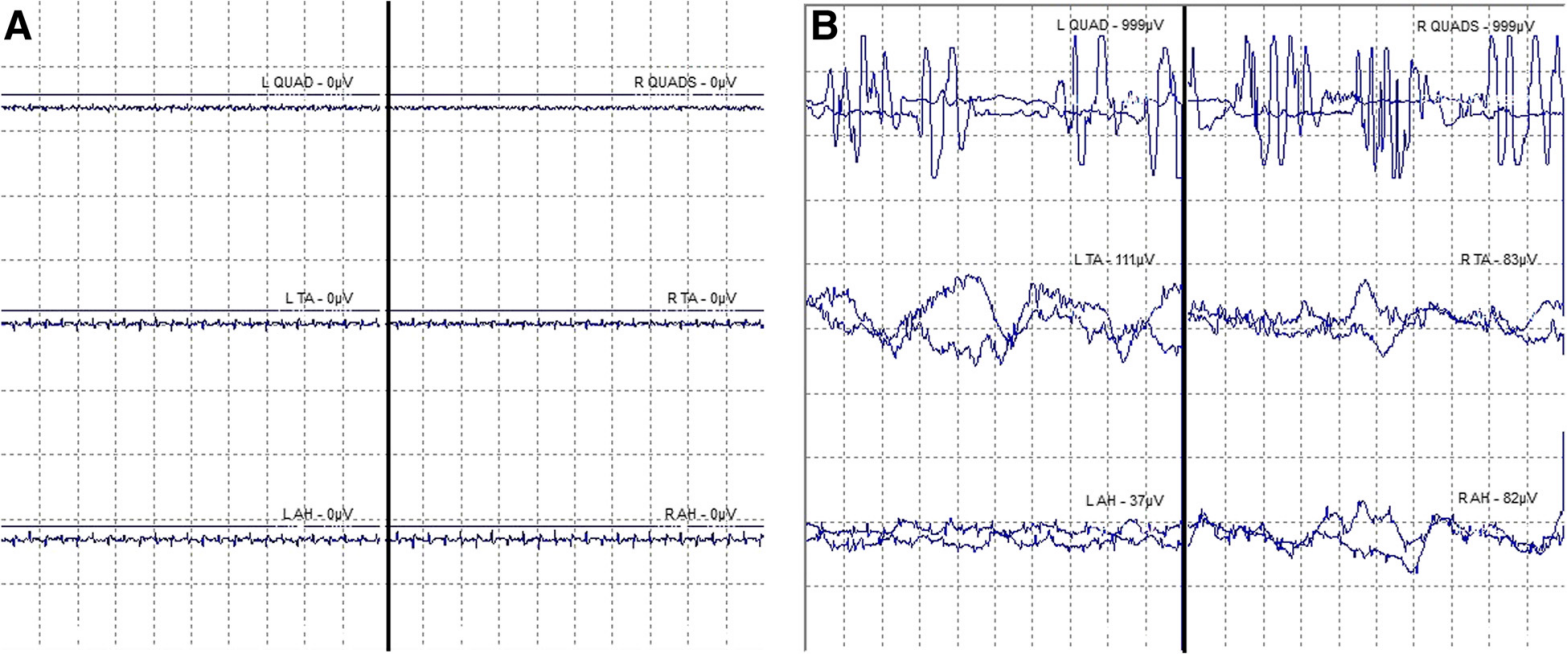
Intraoperative electromyography recordings displaying patient spasms. Baseline intraoperative free-run EMG activity before muscle spasms displaying no muscle activity (**a**), and EMG burst activity during intraoperative leg muscle spasms (**b**). The activity during the muscle spasms is most pronounced in the quadriceps, although all EMGs showed substantial activity at this time. *EMG* electromyography, *QUAD* vastus medialis, *TA* tibialis anterior, *AH* abductor halluces

**Fig. 3:**
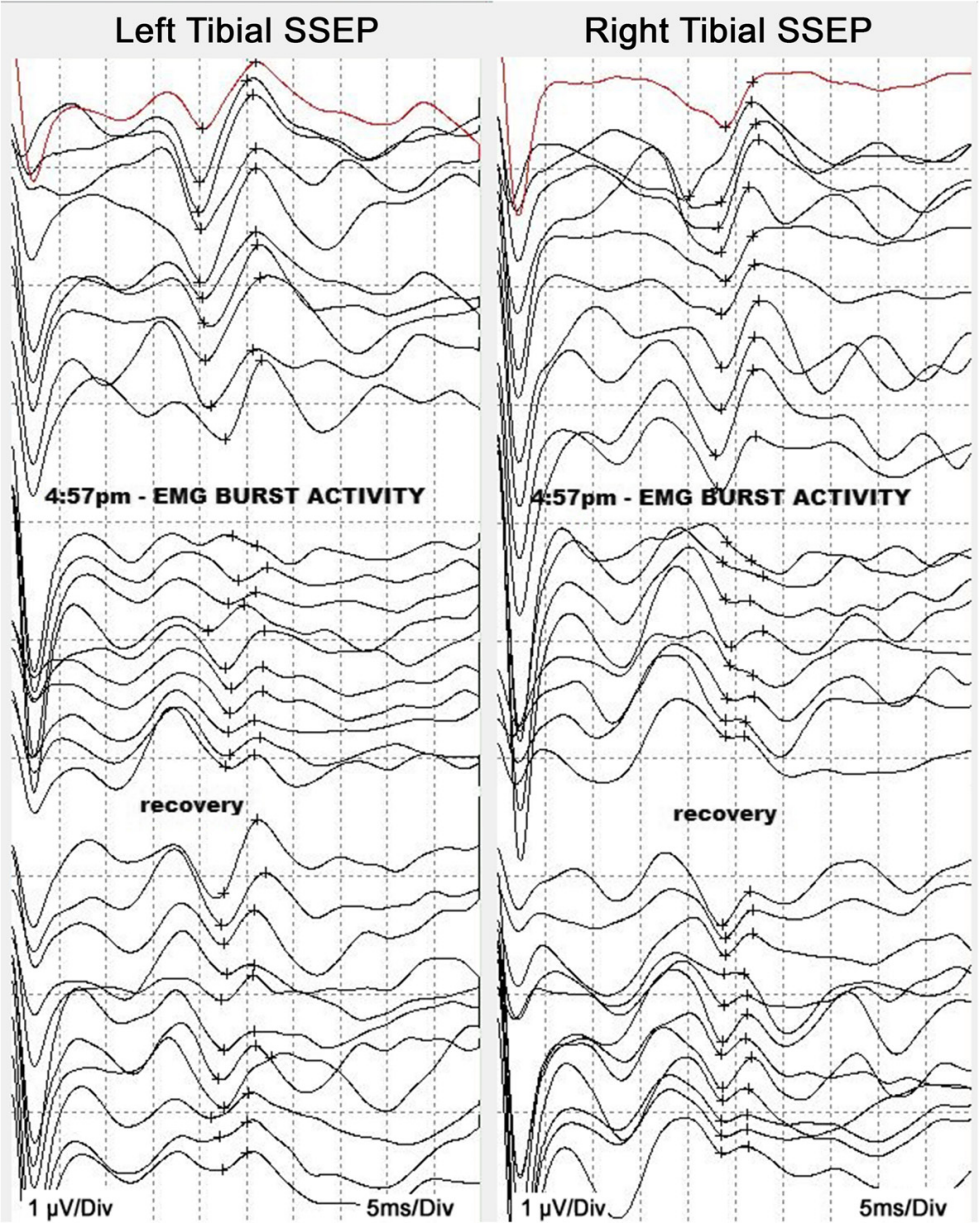
Intraoperative neurophysiological monitoring displaying loss of tibial somatosensory evoked potentials and recovery. Loss of tibial SSEPs during intraoperative leg muscle spasms and partial recovery after decompression; the traces are in descending order of time, with the first trace in *red* as the baseline. The SSEP amplitudes in both tibial responses are dramatically reduced following “EMG burst activity”, as marked between the crosshairs on each trace. It is thought that the muscle spasms led to increased intradiscal pressure and then intraoperative herniation, followed by loss of tibial SSEPs. The “recovery” of SSEPs occurred following decompression of the herniated disc. The last three traces of tibial SSEPs actually look worse, possibly due to the addition of inhalational anesthesia toward the end of the operation. Full clinical recovery of function may be attributed to additional time for the patient to recover from the effects of anesthesia prior to moving her limbs. *EMG* electromyography, *SSEP* somatosensory evoked potential

Following the aforementioned outline to place pedicle screws percutaneously on the left side first, k-wires were positioned and the tap was followed into the L5 left pedicle. At this time, all free-run EMG activity remained quiet (Fig. 2a). While tapping, there was a sudden and profound spasm of our patient’s legs and back that lasted for the duration of 15 seconds, as evidenced by our EMG (Fig. 2b). The frameless guidance system and intraoperative CT imaging confirmed that the tap was accurately positioned in the pedicle. At this time, IONM indicated a loss of all SSEPs in our patient’s lower extremities (Fig. 3). Consideration was given to the possibility of further extrusion of the L5-S1 disc, resulting in a cauda equina compression. A minimal-access tubular approach was immediately taken to expose the previous microlaminotomoy site, where extruded disc material was protruding. Several large disc fragments were extracted, and the dura and neural roots were then visualized. Upon completion of the decompression the SSEPs all began to recover. An interbody device was placed for fusion in standard fashion (with no loose disc fragments remaining), and then the pedicle screws on the right side were placed followed by rod placement with the Sextant system (Medtronic Sofamor Danek USA, Inc., Memphis, TN, USA) A follow-up intraoperative O-arm CT scan was performed, revealing the screws and interbody device to be in excellent position concurrently with no response in the IONM (indicating that the nerve roots were not injured from the pedicle screws). Positioning was confirmed by X-ray 1 week later (Fig. 1b and 1c). Our patient awoke with normal strength and sensation in her lower extremities.

## Discussion

To the best of our knowledge, this is the first reported case of intraoperative disc herniation that occurred while performing a secondary discectomy and fusion. It is clear from our SSEP and EMG recordings that a separate neurologic event occurred, which resulted in cauda equina compression and loss of sensory pathways (Figs. 2 and 3). The finding of disc material herniating through the previous microlaminotomy with several large extruded fragments detected, along with recovery of the sensory pathways upon removal of these fragments, is evidence for the occurrence of a further herniation intraoperatively (Fig. 3). We presume that the spasms that occurred resulted in an increase of intradiscal pressure, which resulted in this further herniation, resulting in compression of the lumbar nerve roots and leading to a loss of SSEPs.

An occurrence of intraoperative spasms can usually be explained by anesthetic lightening, electrocautery stimulation, anatomical changes, or static electrical discharge from a surgical tool. The spasms were unlikely related to anesthetic lightening, because our electroencephalography (EEG) recordings showed our patient to be in deep sleep at the time of spasm. Bipolar electrocautery was not being used at the time of spasm either, eliminating the possible spread of an electrical system as a cause. Additionally, direct irritation of the nerve root from possible anatomical changes was ruled out by the intraoperative CT scan and IONM monitoring, because the frameless stereotaxic guidance system ensured that there was no breach of the pedicle screws into the nerve roots, and there was no neurophysiological response to pedicle screw placement in IONM. When tapping the pedicle, it is possible to misdirect the tap, and it could theoretically damage one or multiple nerve roots, but it was clear that this was not the case in this instance, again as indicated by our postoperative scans showing the pedicle screws in proper placement (Fig. 1b and 1c). Another anatomical possibility is that the slight force used during left pedicle tapping was sufficient enough to exacerbate a reherniation through the previous annulotomy site, considering this was already a mobile segment.

It is also possible that there was a discharge of static electricity from the surgical instrumentation as it was being placed over the k-wire, which resulted in nerve stimulation. The buildup of static electricity on surgical equipment has been recognized for years now [8], however, we found no specific literature reference to lower extremity muscle spasms related to static electrical discharge. An antistatic silicone rubber mat for retaining surgical instruments was used during the operation, but other surgical devices (such as the surgical probe) may still have accumulated in charge during the operation. It is possible that none of the foregoing hypotheses are correct and that another, yet to be appreciated, event occurred.

A recent investigation of 100 consecutive TLIFs revealed that the incidence of minor surgical complication in these procedures was 16 %, and permanent surgical complication was very low [9]. The use of IONM during decompression/reconstruction surgeries is an additional factor that contributes to patient safety and potentially alerts the surgeon if there is a complication. Nonetheless, there is controversy over the usefulness of IONM in degenerative disc surgeries performed in the lumbar spine [10, 11, 12]. It is well understood by surgeons that IONM may not always indicate intraoperative neural deficits because motor evoked potentials or SSEPs can remain unaffected. Such was the case in a recent report on an intraoperative disc herniation that occurred in a 17-year-old patient during posterior spinal fusion for correction of Scheuermann’s kyphosis [13]. However, IONM is still preferred to an intraoperative wake-up test, and is effective in certain spine operations. A case series directed at Athens University Medical School demonstrated the usefulness of IONM in allowing for intraoperative pedicle screw correction, revealing a greater reduction in the number of misdirected pedicle screws and thus neurologic complications when IONM was used [14]. Specifically, it was found that IONM had a predictive value of 98.73 % for indicating misdirection of pedicle screws in posterior thoracolumbar spinal fusion.

In the ongoing dialogue over whether IONM is necessary in spinal decompression and reconstruction procedures, this case demonstrates the significant value of IONM in indicating the onset of an unanticipated and otherwise unrecognized event leading to profound neural compression. If the herniation had occurred intraoperatively at another disc level, without IONM there would have been no indication of its occurrence until postoperative deficits were evident. The ability of IONM to allow us to address this intraoperative herniation allowed for the avoidance of permanent neurologic injury.

## Conclusions

This case presented with an unusual set of circumstances: (1) muscle spasms in the lower extremities during percutaneous lumbar pedicle screw tapping; (2) loss of tibial nerve SSEPs that occurred soon thereafter as well as EMG activation; (3) exploration of the previous microlaminotomy with identification and removal of large extruded fragments; and (4) immediate recovery of tibial SSEPs that correlated with normal neurologic examination postoperatively and quieting of the EMGs.

Neurosurgeons and anesthesiologists should be wary of possible conditions and hazards that can cause a patient to go into spasm during an operation. The likelihood of an intraoperative disc herniation is very small, but the possibility exists. In this case, the use of IONM was deemed critical in addressing a surgical complication rapidly, thus avoiding permanent neurologic injury.

## Consent

Written informed consent was obtained from the patient for publication of this case report and any accompanying images. A copy of the written consent is available for review by the Editor-in-Chief of this journal.

## Abbreviations

CT: computed tomography
EEG: electroencephalography
EMG: electromyography
IONM: intraoperative neurophysiological monitoring
MRI: magnetic resonance imaging
SSEP: somatosensory evoked potential
TLIF: transforaminal lumbar interbody fusion
